# A randomized controlled study of socioeconomic support to enhance tuberculosis prevention and treatment, Peru

**DOI:** 10.2471/BLT.16.170167

**Published:** 2017-02-09

**Authors:** Tom Wingfield, Marco A Tovar, Doug Huff, Delia Boccia, Rosario Montoya, Eric Ramos, Sumona Datta, Matthew J Saunders, James J Lewis, Robert H Gilman, Carlton A Evans

**Affiliations:** a Department of Clinical Infection, Microbiology and Immunology, Institute of Infection and Global Health, Ronald Ross Building, 8 West Derby Street, Liverpool, L69 7BE, England.; b Innovation For Health And Development (IFHAD), Universidad Peruana Cayetano Heredia, Lima, Peru.; c Innovación Por la Salud Y Desarrollo (IPSYD), Asociación Benéfica Prisma, Lima, Peru.; d Department of Infectious Disease Epidemiology, London School of Hygiene & Tropical Medicine, London, England.; e Section of Infectious Diseases and Immunity, Imperial College London, London, England.; f Department of International Health, Johns Hopkins Bloomberg School of Public Health, Baltimore, United States of America.

## Abstract

**Objective:**

To evaluate the impact of socioeconomic support on tuberculosis preventive therapy initiation in household contacts of tuberculosis patients and on treatment success in patients.

**Methods:**

A non-blinded, household-randomized, controlled study was performed between February 2014 and June 2015 in 32 shanty towns in Peru. It included patients being treated for tuberculosis and their household contacts. Households were randomly assigned to either the standard of care provided by Peru’s national tuberculosis programme (control arm) or the same standard of care plus socioeconomic support (intervention arm). Socioeconomic support comprised conditional cash transfers up to 230 United States dollars per household, community meetings and household visits. Rates of tuberculosis preventive therapy initiation and treatment success (i.e. cure or treatment completion) were compared in intervention and control arms.

**Findings:**

Overall, 282 of 312 (90%) households agreed to participate: 135 in the intervention arm and 147 in the control arm. There were 410 contacts younger than 20 years: 43% in the intervention arm initiated tuberculosis preventive therapy versus 25% in the control arm (adjusted odds ratio, aOR: 2.2; 95% confidence interval, CI: 1.1–4.1). An intention-to-treat analysis showed that treatment was successful in 64% (87/135) of patients in the intervention arm versus 53% (78/147) in the control arm (unadjusted OR: 1.6; 95% CI: 1.0–2.6). These improvements were equitable, being independent of household poverty.

**Conclusion:**

A tuberculosis-specific, socioeconomic support intervention increased uptake of tuberculosis preventive therapy and tuberculosis treatment success and is being evaluated in the Community Randomized Evaluation of a Socioeconomic Intervention to Prevent TB (CRESIPT) project.

## Résumé

**Objectif:**

Évaluer l'impact de l'accompagnement socioéconomique sur le commencement du traitement préventif contre la tuberculose par les contacts familiaux des patients atteints de la maladie et sur la réussite du traitement pour les patients.

**Méthodes:**

Une étude contrôlée, non aveugle, à répartition aléatoire des foyers a été réalisée entre février 2014 et juin 2015 dans 32 bidonvilles du Pérou. Elle portait sur des patients traités contre la tuberculose et leurs contacts familiaux. Les foyers ont été choisis de façon aléatoire pour recevoir soit les soins standards prévus par le programme national de lutte contre la tuberculose du Pérou (groupe témoin), soit les mêmes soins standards plus un accompagnement socioéconomique (groupe expérimental). L'accompagnement socioéconomique comprenait des transferts monétaires conditionnels pouvant atteindre 230 dollars des États-Unis par foyer, des visites à domicile et des réunions communautaires. Le taux de commencement du traitement préventif contre la tuberculose et le taux de réussite du traitement (guérison ou achèvement du traitement) ont été comparés entre le groupe expérimental et le groupe témoin.

**Résultats:**

Au total, 282 foyers sur 312 (90%) ont accepté de participer: 135 dans le groupe expérimental et 147 dans le groupe témoin. 410 contacts avaient moins de 20 ans: dans le groupe expérimental, 43% ont commencé un traitement préventif contre la tuberculose, contre 25% dans le groupe témoin (rapport des cotes ajusté (RC): 2,2; intervalle de confiance (IC) de 95%: 1,1-4,1). Une analyse par intention de traiter a montré la réussite du traitement chez 64% (87/135) des patients du groupe expérimental contre 53% (78/147) du groupe témoin (RC non ajusté: 1,6; IC 95%: 1,0-2,6). Ces améliorations étaient équitables et indépendantes de la pauvreté des foyers.

**Conclusion:**

Une intervention d'accompagnement socioéconomique spécifiquement axé sur la tuberculose a permis d'augmenter la prise d'un traitement préventif contre la tuberculose ainsi que la réussite du traitement contre cette maladie. Elle est actuellement évaluée dans le cadre du projet CRESIPT (Community Randomized Evaluation of a Socioeconomic Intervention to Prevent TB).

## Resumen

**Objetivo:**

Evaluar el impacto del apoyo socioeconómico en la iniciación a la terapia preventiva contra la tuberculosis en contactos domésticos de pacientes con tuberculosis, así como en el éxito del tratamiento para los pacientes.

**Métodos:**

Entre febrero de 2014 y junio de 2015, se realizó un estudio controlado, aleatorizado, doméstico y no cegado en 32 barrios bajos de Perú. En este estudio se incluyeron pacientes que estaban siendo tratados contra la tuberculosis y sus contactos domésticos. Los hogares se asignaron de forma aleatoria a la atención estándar ofrecida por el programa nacional contra la tuberculosis de Perú (grupo de control) o bien a la misma atención estándar pero con un apoyo socioeconómico (grupo de intervención). El apoyo socioeconómico consistía en transferencias de efectivo condicionadas de hasta 230 dólares estadounidenses por hogar, visitas domésticas y reuniones comunitarias. Se compararon los grupos de control y de intervención en cuanto a las tasas de iniciación a la terapia preventiva contra la tuberculosis y al éxito del tratamiento (es decir, la cura o la finalización del tratamiento).

**Resultados:**

En general, 282 de 312 (90%) hogares aceptaron participar: 135 en el grupo de intervención y 147 en el grupo de control. Había 410 contactos menores de 20 años: el 43% del grupo de intervención inició la terapia preventiva contra la tuberculosis, frente al 25% del grupo de control (coeficiente de posibilidades ajustado, CPa: 2,2; intervalo de confianza, IC, del 95%: 1,1–4,1). Un análisis de intención de tratar mostró que el tratamiento tuvo éxito en un 64% (87/135) de los pacientes del grupo de intervención, frente a un 53% (78/147) de los pacientes del grupo de control (CP no ajustado: 1,6; IC del 95%: 1,0–2,6). Estas mejoras fueron equitativas, independientemente de la pobreza del hogar.

**Conclusión:**

Una intervención de apoyo socioeconómico específica para la tuberculosis aumentó la aceptación de la terapia preventiva contra la tuberculosis y el éxito del tratamiento, y se está evaluando en el proyecto *Community Randomized Evaluation of a Socioeconomic Intervention to Prevent TB* (CRESIPT – Evaluación Aleatoria Comunitaria de una Intervención Socioeconómica para Prevenir la TB).

## ملخص

**الغرض:**

تقييم مدى تأثير الدعم الاجتماعي الاقتصادي على طرح العلاج الوقائي من السل لدى الأُسر التي تتعامل مع مرضى السل ومدى تأثيره على نجاح علاج المرضى.

**الطريقة:**

تم إجراء دراسة غير عمياء، عشوائية قائمة على الأُسر، مضبطة في الفترة ما بين فبراير/شباط 2014 إلى يونيو/حزيران 2015 في 32 حيًا فقيرًا في بيرو. وشملت هذه الدراسة المرضى الذين خضعوا للعلاج من السل وأفراد أسرتهم. وتم إسناد الأُسر بشكلٍ عشوائي إما إلى معيار الرعاية التي يوفرها البرنامج القومي لمواجهة السل في بيرو (الشِق الخاص بالرقابة) أو نفس المعيار الخاص بالرعاية بالإضافة إلى الدعم الاقتصادي الاجتماعي (الشِق الخاص بالتدخل). ويتألف الدعم الاقتصادي الاجتماعي من الحوالات النقدية الشرطية التي تصل إلى 230 دولارًا أمريكيًا لكل أسرة، والزيارات المنزلية، واللقاءات المجتمعية. وتمت مقارنة معدلات مبادرة العلاج الوقائي من السل ونجاح العلاج (أي إكمال العلاج أو تحقيق الشفاء) في الشقين المتعلقين بالرقابة والتدخل.

**النتائج:**

إجمالاً، وافقت 282 من أصل 312 (‏90‏%) أسرة على المشاركة: 135 في الشِق الخاص بالتدخل، و147 في الشق الخاص بالرقابة. وكان هناك حوالي 410 أِشخاص محتكين بالمرضى ممن هم أقل من 20 سنة: وبدأ 43‏% منهم في الشِق الخاص بالتدخل في العلاج الوقائي من السل مقابل 25‏% منهم في الشق الخاص بالتحكم (بنسبة احتمال معدّلة تبلغ: 2.2؛ بنسبة أرجحية مقدارها ‏95‏%: 1.1–4.1). وأوضح تحليل لقصد العلاج أن العلاج سجل نجاحًا لدى 64‏% من المرضى (87/135) في الشِق الخاص بالتدخل مقابل 53‏% (78/147) في الشِق الخاص بالرقابة (نسبة احتمال غير معدّلة تبلغ: 1.6؛ ‏95‏% كمقدار لنسبة الأرجحية: 1.0–2.6). وكانت تلك التحسينات عادلة بالنظر إلى عدم ارتباطها بفقر الأُسر.

**الاستنتاج:**

ساهم الدعم الاجتماعي الاقتصادي المخصص لمواجهة داء السل في زيادة استيعاب العلاج الوقائي لداء السل ونجاح علاج داء السل، ويجري تقييمه في إطار التقييم المجتمعي العشوائي لمشروع الدعم الاجتماعي الاقتصادي لمواجهة داء السل (CRESIPT). ****

## 摘要

**目的:**

旨在评估社会经济支持对在结核病患者家庭接触者中发起的结核病预防性治疗的影响以及对患者治疗成功的影响。

**方法:**

于 2014 年 2 月至 2015 年 6 月期间，在秘鲁的 32 个贫民窟开展了一项非盲法家庭随机型对照研究。 该项研究包括因结核病接受治疗的患者及其家庭接触者。所有家庭随机分为接受秘鲁国家结核病项目提供的标准护理的标准护理组(对照组)，和标准护理加社会经济支持组（干预组）。社会经济支持包括每户高达 230 美元的有条件现金转移支付、家庭探望及社区会议。 对干预组及对照组的结核病预防性治疗的发起率及患者治疗成功率（即，治愈或完成治疗）进行了对比。

**结果:**

312 户家庭中总计有 282 (90%) 户家庭同意参加：135 户家庭为干预组，147 户家庭为对照组。其中有 410 名接触者小于 20 岁： 干预组的结核病预防性治疗发起率为 43%，对照组为 25%（调整后比值比，OR： 2.2；95% 置信区间，CI： 1.1–4.1)。 一项意向治疗分析显示，干预组患者的治疗成功率为 64% (87/135)，对照组为 53% (78/147) （调整后 OR： 1.6; 95% CI: 1.0–2.6)。 这些改进均衡分布，与家庭贫困程度无关。

**结论:**

结核病专项社会经济支持干预提高了结核病预防性治疗的接受率及结核病的治疗成功率，目前正在接受结核病社会经济干预的社区随机化评估 (CRESIPT) 项目的评估。

## Резюме

**Цель:**

Оценить, как влияет социально-экономическая поддержка на начало профилактического противотуберкулезного лечения членами семьи, контактирующими с больным, а также оценить вклад такой поддержки в успех лечения.

**Методы:**

Открытое контролируемое исследование с рандомизацией семей было проведено в период между февралем 2014 года и июнем 2015 года в 32 трущобных поселениях в Перу. В исследование включались пациенты, получающие лечение от туберкулеза, и члены их семей, контактировавшие с ними. Семьям случайным образом назначали либо стандартное лечение, положенное в соответствии с национальной противотуберкулезной программой, осуществляемой в Перу (контрольная группа), либо такое же стандартное лечение в сочетании с социально-экономической поддержкой (экспериментальная группа). Социально-экономическая поддержка включала выдачу денежных пособий при соблюдении определенных условий (в сумме до 230 долларов США на семью), посещение семей и собрания общины. В контрольной группе и в экспериментальной группе сравнивались доли участников, начавших профилактическое противотуберкулезное лечение и успешно его завершивших (под успешным завершением подразумевалось излечение или завершение курса лечения).

**Результаты:**

Принять участие в исследовании согласились 282 семьи из 312 (90%). В экспериментальную группу вошли 135 семей, и 147 семей составили контрольную группу. В исследовании участвовали 410 контактирующих лиц в возрасте моложе 20 лет. В экспериментальной группе профилактическое противотуберкулезное лечение начали осуществлять 43% участников против 25% в контрольной группе (скорректированное отношение шансов, ОШ: 2,2; 95%-й доверительный интервал, ДИ: 1,1–4,2). Согласно результатам анализа по всем рандомизированным участникам, лечение было успешным в 64% случаев (87 из 135 семей) в экспериментальной группе и в 53% случаев (78 из 147 семей) в контрольной группе (нескорректированное ОШ: 1,6; 95%-й ДИ: 1,0–2,6). Улучшения наблюдались в равной мере и не зависели от того, насколько бедной была семья.

**Вывод:**

Социально-экономическая поддержка, специально ориентированная на помощь туберкулезным больным, увеличивает использование профилактического лечения и шанс успешного противотуберкулезного лечения и оценивается в рамках проекта оценки социально-экономических вмешательств для предотвращения туберкулеза с рандомизацией по сообществам (Community Randomized Evaluation of a Socioeconomic Intervention to Prevent TB, CRESIPT).

## Introduction

An estimated one third of the world’s population has latent tuberculosis infection and in 2015 10.4 million people developed tuberculosis disease.^1^ Those at the highest risk of tuberculosis include the household contacts of patients with the disease and people living in poverty.^2^ Trials have shown that preventive therapy decreases the risk of progression to tuberculosis disease by 60 to 90%.^2^^–^^4^ Nevertheless, globally the impact of preventive therapy on tuberculosis control is limited because people with a latent tuberculosis infection are seldom identified^5^ and, therefore, seldom take preventive therapy.^6^^–^^9^ In addition, many people have difficulty adhering to treatment^7^^,^^8^^,^^10^ and tuberculosis patients who do not take adequate treatment are more likely to experience adverse outcomes, such as treatment failure, tuberculosis recurrence and death.^11^ They are also more likely to transmit the infection, especially to household contacts^12^ and to develop multidrug-resistant tuberculosis,^13^ an increasing global public health threat.^5^

The current, predominantly biomedical approach to tuberculosis control is not reducing disease incidence to the level required to eliminate tuberculosis envisioned in the World Health Organization’s (WHO) End TB Strategy.^14^^,^^15^ Increasing access to tuberculosis preventive therapy and treatment is likely to improve disease prevention and treatment success but requires strategies complementary to biomedical care, including socioeconomic support. Interventions such as conditional cash transfers can help improve people’s capacity to manage social and financial risks.^16^^–^^23^ Although socioeconomic interventions are common in the treatment of human immunodeficiency virus infection (HIV) and acquired immune deficiency syndrome (AIDS) and in maternal health,^24^^,^^25^ little is known about their impact on tuberculosis care or prevention.^16^^,^^18^^,^^19^^,^^26^

Our research group in Peru, Innovation for Health and Development, has been funded to undertake the Community Randomized Evaluation of a Socioeconomic Intervention to Prevent TB (CRESIPT) project. The planning, design and economic impact of the intervention have been described previously.^27^^,^^28^ Here we report the final results of the initial phase of CRESIPT, which involved a household-randomized, controlled study that evaluated the impact of tuberculosis-specific socioeconomic support on the initiation of tuberculosis preventive therapy and on tuberculosis treatment success. In addition, we describe the refinement of this intervention used in CRESIPT.

## Methods

**Fig. 1:**
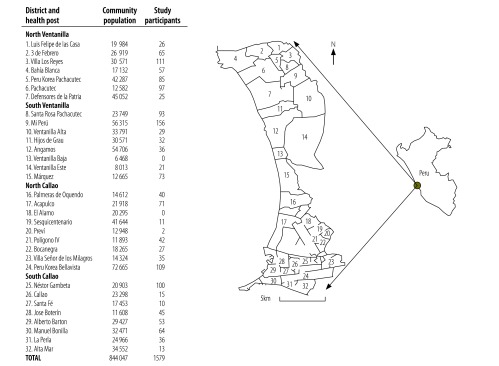
Study area and participants, study of the effect of socioeconomic support on tuberculosis prevention and treatment, Peru, 2014–2015

Note: The map of Peru is from Wikimedia Commons and is licensed under the Creative Commons Attribution-Share Alike 3.0 Unported license.^33^

The study evaluated the impact of a socioeconomic support intervention – described in Box 1 – in 32 contiguous shanty towns in Callao, Peru, the northern, coastal extension of the capital Lima (Fig. 1). The Province of Callao has a population of 1 million, considerable poverty and zones with high levels of drug addiction and gun crime. The annual tuberculosis case notification rate in 2014 was 123 new cases per 100 000 population, the highest rate in the country.^34^

Box 1 **Description of the socioeconomic support intervention for tuberculosis prevention and treatment, Peru, 2014–2015**

The socioeconomic support intervention comprised an integrated package of social and economic support.^27^ The intervention targeted outcomes on the tuberculosis causal pathway and promoted equitable access to tuberculosis programme activities, including: (i) screening for tuberculosis in contacts of patients; (ii) the initiation of tuberculosis preventive therapy and completion of tuberculosis treatment; and (iii) engagement with social support activities.

Social support comprised household visits and participatory community meetings that aimed to provide information and mutual support, empowerment and reduce the stigma of tuberculosis. Household visits were made shortly after the patient commenced treatment and involved providing education on tuberculosis transmission, treatment and preventive therapy and on household finances. Community meetings took place monthly and were each attended by around 15 patients and their household contacts. They cost around 189 United States dollars (US$) each (approximately US$ 13 per patient per meeting).^27^ The meetings reinforced the educational themes of the household visits and established tuberculosis clubs, in which participants could share their tuberculosis-related experiences in a mutually supportive group (to be reported elsewhere). All household members were invited and encouraged to participate in household visits and community meetings.

Economic support comprised making conditional cash transfers throughout treatment to defray average household tuberculosis-associated costs, thereby reducing risk factors for tuberculosis while also incentivizing and enabling care. Economic support was designed to ensure direct out-of-pocket expenses would be completely defrayed for patients who received all conditional cash transfers. Previously, such direct out-of-pocket expenses had been found to be 10% of annual household income in the study setting,^29^ equivalent to approximately US$ 230. We hypothesized that defraying these direct expenses would decrease the tuberculosis-affected household’s financial burden, decrease the likelihood of incurring catastrophic costs and, when combined with integrated social support, enhance access to tuberculosis care and improve tuberculosis outcomes. During the planning of the intervention it was estimated that, if the intervention were implemented nationally, the budget of the Peruvian National Tuberculosis Programme would have to increase by approximately 15% per patient.^29^ Focus group discussions with key stakeholders suggested that such an increase was locally appropriate and affordable.^27^^,^^29^^,^^30^ Moreover, a review of the relevant literature suggested that interventions that increased the per-patient cost of a tuberculosis programme budget by 50% or less and that reduced the incidence of tuberculosis by at least one third were likely to be cost-effective and sustainable.^31^^,^^32^

The socioeconomic support intervention was informed by the findings of our group’s Innovative Socioeconomic Interventions Against TB (ISIAT) study,^6^ two systematic reviews of cash-transfer interventions,^16^^,^^27^ expert consultations^18^ and feedback from civil society and leaders of the Peruvian National Tuberculosis Programme.^27^

The study included the households of patients starting treatment for tuberculosis disease administered by the Peruvian National Tuberculosis Programme. The invitation to participate was accompanied by a written informed consent form that explained the randomization process. Patients completed the form on the household’s behalf. For minors, a parent or guardian gave consent with the patient’s assent. We only included consenting households. Individuals reported by the patient during a household visit to have been in the same house as the patient for over 6 hours per week in the 2 weeks before tuberculosis was diagnosed, were identified and validated and are henceforth described as contacts. Contacts declared or discovered following randomization (but not during initial recruitment) were not included in the analysis and were not invited to participate in the study.

Subsequently, patients’ households were randomly assigned on a 1:1 ratio to either: (i) the control arm, in which households received the standard of care provided by the Peruvian National TB Programme; or (ii) the intervention arm, in which households additionally received the integrated socioeconomic support package. Randomization was performed using random number tables, which generated individual household randomization sequences for each health post. Once a patient gave informed consent, a project nurse opened a numbered, sealed envelope that contained the study allocation and revealed the allocation to the patient. It was not feasible to blind households or the research team to the allocation. However, staff members from the national tuberculosis programme were not informed and were generally unaware of a household’s allocation but they were not confirmed as being blinded.

Data on health, well-being and sociodemographic characteristics, including height, weight, body mass index and socioeconomic position, were collected using a locally validated questionnaire at baseline (i.e. at the start of tuberculosis treatment) and again 24 weeks later, or 28 weeks later if treatment was prolonged, due, for example, to suboptimal treatment adherence.^6^^,^^27^^,^^29^

### Treatment

For the contacts of patients with pulmonary tuberculosis that was not caused by multidrug-resistant bacteria, Peruvian National Tuberculosis Programme guidelines, which were applied throughout the study, recommended that preventive therapy should be: (i) provided for all contacts younger than 5 years, unless the contact is known to have previously had tuberculosis disease, without tuberculin skin testing; and (ii) considered for all contacts aged 5 to 19 years with a positive tuberculin skin test result.^29^ However, tuberculin was generally unavailable throughout the study. Preventive therapy consisted of a 6-month course of daily isoniazid, which contacts collected weekly from health posts and took unsupervised at home.^29^ Data on preventive therapy initiation, adherence and completion were obtained from the Peruvian National TB Programme records and included the number of weeks of preventive therapy collected (hereafter defined as preventive therapy taken) from the health post for each household contact.

The Peruvian National TB Programme offered free tuberculosis diagnostic testing to all people with symptoms suggestive of tuberculosis. If diagnosed with the disease, they received free anti-tuberculosis treatment at the health post under the directly-observed-treatment (DOTS) strategy.^29^ In addition, all patients, regardless of their allocation, were offered a sputum test with Xpert MTB/RIF (Cepheid, Sunnyvale, United States of America) at our research laboratory for rapid rifampicin susceptibility testing – this test was not otherwise routinely available.

### Outcomes

The primary study outcome was initiation of tuberculosis preventive therapy by a contact younger than 20 years who was available for follow-up. The secondary study outcome was successful tuberculosis treatment of a patient with the disease, which was assessed on an intention-to-treat basis and included patients with unknown outcomes. Successful tuberculosis treatment was defined as either a cure or completed treatment. In accordance with WHO definitions,^1^ the Peruvian National TB Programme guidelines regarded patients with bacteriologically confirmed, drug-susceptible tuberculosis at diagnosis as having been cured if they: (i) completed treatment; (ii) had a negative sputum smear test result during the final month of treatment; and (iii) had received a favourable clinical assessment by a national programme physician who had evaluated their symptoms, performed an examination, weighed them and, when necessary, carried out chest radiography and blood tests.^29^ Patients were regarded as having completed tuberculosis treatment if they completed the treatment course without evidence of failure, even if they did not undergo the required sputum testing or physician review. Other outcomes consistent with WHO guidance were: (i) death due to any cause before or during tuberculosis treatment; (ii) treatment failure (i.e. positive sputum microscopy or culture findings after 5 months of treatment or later); and (iii) lost to follow-up, which included patients whose treatment was interrupted for at least 30 consecutive days or who discontinued treatment having been treated for less than 30 days – this is shorter than the 2-month or longer interruption in WHO’s definition. Treatment outcome data were collected from each patient’s treatment card at the final follow-up in collaboration with the Peruvian National TB Programme and were not influenced by this research. Outcomes could not be assessed in patients whose treatment outcome had not been assigned, such as those who had been transferred to another treatment unit and those who were still on treatment at the 28-week follow-up interview (e.g. patients with multidrug-resistant tuberculosis, who are often treated for 24 months). The study was approved by the ethics committees of the Regional Ministry of Health in Callao, Asociación Benéfica Prisma in Peru, and Imperial College London, United Kingdom of Great Britain and Northern Ireland.

### Statistical analysis

Sample size calculations indicated that a study including 400 contacts would have 80% statistical power to detect a 50% increase in the primary outcome in intervention households compared with control households with a two-sided 5% level of significance.^6^ We assessed differences in treatment success and preventive therapy initiation rates between the study groups using univariable logistic regression analysis and, in the case of treatment success, also by multivariable logistic regression analysis to adjust for household clustering. The level of household poverty was determined by combining socioeconomic variables into a composite index using principal component analysis, as previously described.^29^ The significance of the difference in the duration of preventive therapy taken by contacts younger than 20 years in intervention and control households was assessed using the Mann–Whitney *U* test and by time-to-event analysis, which generated an unadjusted log-rank *P*-value.

## Results

**Fig. 2:**
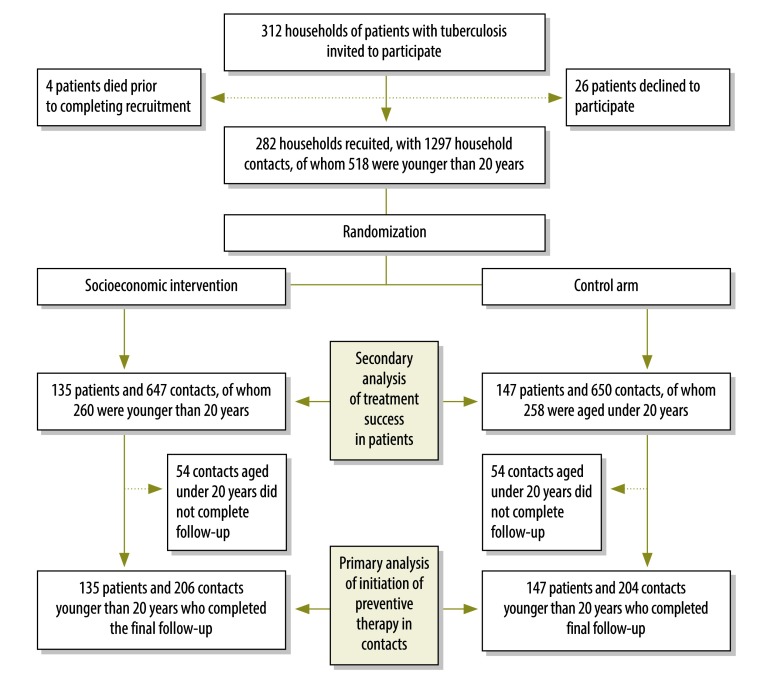
Flowchart, study of the effect of socioeconomic support on tuberculosis prevention and treatment, Peru, 2014–2015

Recruitment commenced on 10 February 2014, the target sample size was reached on 14 August 2014 and follow-up was completed on 1 June 2015. In total, we invited 312 households of patients with tuberculosis to participate and we recruited 90% (282/312), of which we randomized 135 households to the intervention arm and 147 to the control arm. Overall, 9% (24/282) of patients had multidrug-resistant tuberculosis, none of whom completed treatment during the study. Patients from the 282 recruited households had a total of 1297 contacts (mean: 4.6 contacts per household). Of the contacts, 40% (518/1297) were younger than 20 years and 79% (410/518) of this age group completed follow-up (Fig. 2). There was no substantive imbalance between households randomized to intervention or control arms in any sociodemographic characteristic (Table 1).

**Table 1:** Baseline characteristics, study of the effect of socioeconomic support on tuberculosis prevention and treatment, Peru, 2014–2015

Characteristics	Intervention households (*n* = 135)	Control households (*n* = 147)	All households (*n* = 282)
**All household contacts**			
Number of contacts identified per household, mean (SD)	4.9 (2.9)	4.4 (2.9)	4.6 (2.9)
Number of contacts aged < 20 years identified per household, mean (SD)	1.9 (1.7)	1.7 (1.7)	1.8 (1.7)
**Contacts aged < 20 years (*n* = 518)**			
Age in years, median (IQR)	9.1 (4.0–15)	9.0 (4.0–14)	9.1 (4.0–14)
Male sex, % (95% CI)	52 (46–58)	53 (47–60)	53 (49–57)
**Patients (*n* = 282)**			
Age in years, median (IQR)	30 (21–45)	28 (20–43)	28 (21–44)
Male sex, % (95% CI)	64 (55–72)	60 (52–68)	62 (56–67)
Completed secondary school, % (95% CI)	27 (20–35)	37 (29–45)	32 (27–38)
Unemployed before diagnosis, % (95% CI)	36 (28–44)	35 (27–43)	36 (30–41)
Number of days went to bed hungry in past month (i.e. food insecurity), mean (95% CI)	1.8 (1.1–2.5)	1.5 (0.9–2.1)	1.6 (1.2–2.1)
Sputum smear-positive,^a^ % (95% CI)	71 (63–79)	68 (60–76)	70 (64–75)
Isoniazid-resistant tuberculosis only, % (95% CI)	6.7 (2.4–11)	8.2 (3.7–13)	7.4 (4.4–11)
MDR-TB, % (95% CI)	6.7 (2–11)	10.2 (5–15)	8.5 (5–12)
HIV-positive, % (95% CI)	3.7 (0.48–6.9)	5.4 (1.7–9.2)	4.6 (2.1–7.1)
Previous tuberculosis episode, % (95% CI)	18 (11–25)	27 (20–35)	23 (18–28)
Body mass index in kg/m^2^, mean (95% CI)	22 (21–23)	22 (21–22)	22 (21–22)
**Households (*n* = 282)**			
Monthly household income in Peruvian soles, mean (95% CI)	1190 (1071–1309)	1271 (1127–1415)	1231 (1138–1325)
Number of people per room (i.e. crowding), mean (95% CI)	1.9 (1.7–2.1)	2.0 (1.8–2.2)	2.0 (1.8–2.1)
Poverty group,^b^ % (95% CI)			
Poorest tercile	41 (32–49)	38 (30–46)	39 (34–45)
Poor tercile	30 (23–38)	35 (27–42)	33 (27–38)
Less-poor tercile	29 (21–37)	27 (20–34)	28 (23–33)

CI: confidence interval; HIV: human immunodeficiency virus; IQR: interquartile range; MDR-TB: multidrug-resistant tuberculosis; SD: standard deviation.

^a^ A sputum smear test result was defined as positive if acid alcohol-fast bacilli were observed by the Peruvian National Tuberculosis Programme reference laboratory or by our research team’s laboratory in a sputum sample obtained before tuberculosis treatment.

^b^ The level of household poverty was determined by combining socioeconomic variables into a composite index using principal component analysis, as previously described.^29^

During the study, 90% (122/135) of households in the intervention arm received at least one conditional cash transfer. A total of 890 conditional cash transfers were made (i.e. 80% of all possible conditional cash transfers) – the average total received per household was 520 Peruvian soles (186 United States dollars, US$) out of a maximum available per household of 640 Peruvian soles (US$ 230).^27^^,^^29^

The proportion of contacts younger than 20 years who initiated tuberculosis preventive therapy was 44% (91/206) in the intervention arm and 26% (53/204) in the control arm. The difference was significant, both in the univariable analysis (odds ratio, OR: 2.2; 95% confidence interval, CI: 1.4–3.3) and the multivariable analysis (adjusted odds ratio, aOR: 2.2; 95% CI: 1.1–4.1), which adjusted for household clustering. In the intention-to-treat analysis of treatment success in patients, the success rate was 64% (87/135) in the intervention arm and 53% (78/147) in the control arm. The difference was significant in the univariable analysis (OR: 1.6; 95% CI: 1.0–2.6). An adjusted analysis was not relevant because there was only one patient per household. In addition, the proportion of patients from intervention households who were cured was significantly greater than the proportion from control households: 53% (71/135) versus 37% (55/147), respectively (*P* = 0.02). Details of the proportions who were cured or achieved other treatment outcomes as defined by WHO are reported in Table 2.

**Table 2:** Treatment outcomes, by study arm and household poverty, study of the effect of socioeconomic support on tuberculosis prevention and treatment, Peru, 2014–2015

Tuberculosis treatment outcome^a^	All households (*n* = 282)		Intervention households (*n* = 135)		Control households (*n* = 147)			Less-poor households^b^ (*n* = 187)					Poorer households^b^ (*n* = 95)			
								Intervention households (*n* = 86)		Control households (*n* = 101)			Intervention households (*n* = 49)		Control households (*n* = 46)	
	No.	% (95% CI)	No.	% (95% CI)	No.	% (95% CI)		No.	% (95% CI)	No.	% (95% CI)		No.	% (95% CI)	No.	% (95% CI)
Cured	126	45 (39–51)	71	53 (44–61)	55	38 (30–45)		39	46 (35–56)	37	37 (27–46)		32	66 (51–79)	18	39 (24–54)
Treatment completed	39	14 (9.8–18)	16	12 (6.3–17)	23	16 (9.7–22)		9	11 (3.9–17)	13	13 (6.2–20)		7	15 (4.1–24)	10	22 (9.4–34)
Treatment failed	1	0.5 (0–1.5)	0	0 (0)	1	0.5 (0–1.1)		0	0 (0)	0	0 (0)		0	0 (0)	1	2 (0–6.6)
Died	11	4.0 (1.6–6.2)	5	4.0 (0.48–6.9)	6	4.0 (0.84–7.3)		4	5.0 (0.11–9.2)	4	4.0 (0.9–7.8)		1	2 (0–6.1)	2	4 (0–11)
Lost to follow-up	48	17 (13–21)	22	16 (10–23)	26	18 (11–24)		18	21 (12–30)	20	20 (12–28)		4	8 (2.2–16)	6	13 (2.9–23)
Not evaluated	57	20 (15–25)	21	16 (9.4–22)	36	25 (17–32)		16	18 (10–27)	27	27 (18–36)		5	10 (1.4–19)	9	20 (7.7–31)

CI: confidence interval.

^a^ Treatment outcomes were those recorded by the Peruvian National Tuberculosis Program in line with World Health Organization guidance.^1^

^b^ Poorer households included the poorest tercile of households and less-poor households comprised all remaining households.

**Fig. 3:**
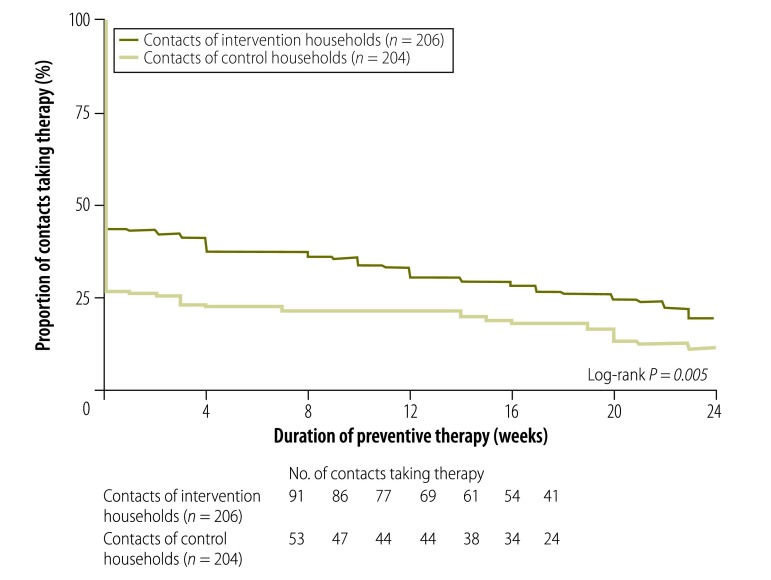
Duration of tuberculosis preventive therapy taken by contacts of patients, study of the effect of socioeconomic support on tuberculosis prevention and treatment, Peru, 2014–2015

The greater use of preventive therapy by contacts younger than 20 years in the intervention arm was maintained throughout the recommended 24 weeks of treatment. Among those who initiated preventive therapy, the mean duration of treatment was similar in intervention and control arms: 18 weeks (standard deviation, SD: 7.7) versus 18 weeks (SD: 7.8), respectively (*P* = 0.9). Consequently, because more contacts initiated tuberculosis preventive therapy in the intervention arm, the mean duration of preventive therapy was significantly longer in the intervention than the control arm: 7.8 weeks (SD: 10) versus 4.8 weeks (SD: 8.9), respectively (*P* = 0.002). Time-to-event analysis confirmed that the intervention was associated with greater overall preventive therapy initiation (log-rank *P* = 0.005; Fig. 3). As the study sample size was selected to test for the effect of the intervention on the whole study population, the study did not have sufficient statistical power to test for effects in subgroups. Thus, although the rate of preventive therapy completion was almost double in the intervention arm (20%; 95% CI: 14–25) than the control arm (12%; 95% CI: 7–16), the difference in this minority of the study population was significant only in the univariable analysis (OR: 1.9; 95% CI: 1.1–3.2) but not in the adjusted analysis (aOR: 1.9; 95% CI: 0.78–4.5).

**Fig. 4:**
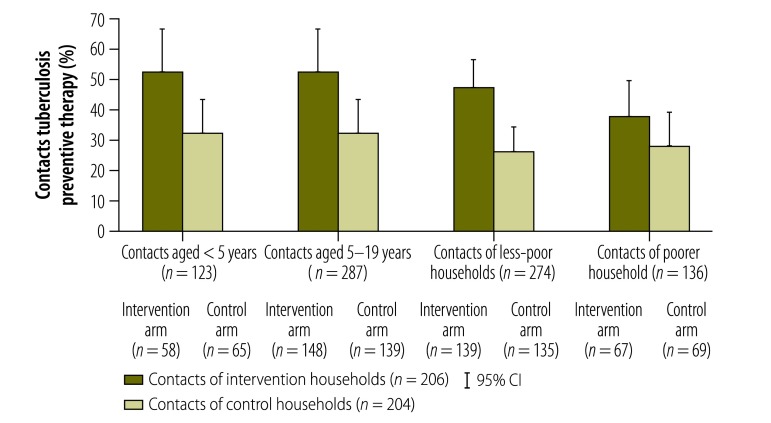
Initiation of tuberculosis preventive therapy by contacts of patients, by study arm, age and household poverty, study of the effect of socioeconomic support on tuberculosis prevention and treatment, Peru, 2014–2015

To assess the equity of the intervention, we compared study outcomes in the most and least vulnerable subpopulations. We compared treatment success and preventive therapy initiation rates in the poorest tercile of the population with the remaining population and compared preventive therapy initiation in child contacts younger than 5 years with contacts aged 5 to 19 years. Table 2 demonstrates that the intervention was associated with an increase in the treatment success rate in both poorer and less-poor subgroups and Fig. 4 shows it was associated with an increase in preventive therapy initiation in poorer and less-poor subgroups and in younger and older contact age groups. Furthermore, the intervention significantly increased preventive therapy initiation in contacts younger than 5 years (aOR: 2.2; 95% CI: 1.1–4.2) and in the poorest tercile (aOR: 2.2; 95% CI: 1.1–4.1). After adjusting for poverty group, the intervention was associated with a nonsignificant trend towards a greater likelihood of treatment success (aOR: 1.7; *P* = 0.07).

CI: confidence interval.

Note: Poorer households included the poorest tercile of households and less-poor households comprised all remaining households.

## Discussion

Previous assessments of interventions for improving tuberculosis prevention or treatment adherence have been limited by a lack of randomization, by small sample sizes or by being conducted in high-resource settings within restricted patient groups, such as HIV-infected people,^35^ homeless people,^36^ migrants^37^ or injecting drug users.^2^^,^^38^ Recent systematic reviews concluded there was no evidence that incentives, including cash transfers, improved tuberculosis preventive therapy completion rates^39^ and there was little evidence to guide WHO recommendations on the implementation and scale-up of tuberculosis-specific, socioeconomic support in resource-constrained settings.^40^ Our study, which found that a tuberculosis-specific, socioeconomic support intervention increased both the uptake of preventive therapy and the success of treatment, helps to fill this evidence gap.^6^^,^^41^

The management of household contacts of tuberculosis patients has been complicated by the current worldwide shortage of tuberculin and the expense, technical complexity and lack of availability of commercial interferon-gamma release assays.^42^ Despite the presence of these obstacles in Peru, our socioeconomic support intervention approximately doubled the tuberculosis preventive therapy initiation rate. Moreover, because the protective effect of preventive therapy increases with its duration,^3^^,^^4^ our finding that the intervention increased the number of weeks of tuberculosis preventive therapy taken is important, given that nonadherence is common,^8^^,^^43^^,^^44^ and could decrease the rate of secondary tuberculosis disease. It is encouraging that the intervention also increased treatment initiation in younger contacts and contacts from poorer households, which suggests that its effect was equitable across age and social groups.

Nevertheless, although completion of 24 weeks of preventive therapy was nearly doubled in contacts from supported households, this increase was not statistically significant. The possible reasons are: (i) only a small number of contacts completed preventive therapy in each study arm and the study was not powered to assess this outcome; (ii) conditional cash transfers were not given monthly for adherence to preventive therapy– they were made only when all eligible household contacts had completed therapy; and (iii) the cash transfers were found not to completely defray direct out-of-pocket expenses because the financial burden of tuberculosis was high for households, as reported previously.^27^^,^^45^ Subsequently, in the CRESIPT study, economic support was increased to completely mitigate direct expenses and monthly conditional cash transfers were introduced for household contacts.

Our study provides evidence supporting WHO’s End TB Strategy, which calls for the existing biomedical paradigm of tuberculosis control to be supplemented by socioeconomic support interventions that address poverty and the other social factors principally responsible for the global tuberculosis epidemic.^14^ In addition to conditional cash transfers, which reduced food insecurity^28^ and improved access to health care, our intervention also involved household visits and community meetings that provided education and information, helped reduce stigma and were empowering – a lack of knowledge about tuberculosis, being female and being marginalized are all risk factors for nonadherence to preventive therapy.^46^ Although our study did not have the power to differentiate the effect of social and economic support, it has been reported that conditional cash transfers alone, without educational or social support, had only a limited impact on HIV-related outcomes.^24^

Our study had several limitations. First, the intention-to-treat analysis did not include treatment outcomes in patients still taking treatment at the final, 28-week follow-up, such as those with multidrug-resistant tuberculosis. Consequently, the proportion of patients whose treatment was successful was probably underestimated in both intervention and, perhaps to a greater extent, control households. However, the majority of our patients were HIV-negative, had drug-susceptible tuberculosis and should have been able to complete treatment by 28 weeks unless it was interrupted. Second, some households may have exaggerated the number of contacts to gain higher cash transfers. However, the number of contacts per household was similar for intervention and control households. Moreover, financial incentives were provided to households rather than individuals and only contacts declared before randomization and confirmed at a household visit were included. Third, patients and the study team were not blinded to the intervention and, in addition, a final conditional cash transfer was made to households in which the patient was cured and contacts completed preventive therapy. As a result, patients in the intervention group may have been more likely to attend their local health post to request confirmation of a cure. Nevertheless, the study team did not encourage staff from the Peruvian National TB Programme to ask patients to confirm they had been cured and patients themselves, in feedback, reported that seeking confirmation was an empowering element of the intervention.^27^ Furthermore, contacts' initiation of preventive therapy and duration of preventive therapy taken was based on the number of weeks of isoniazid tablets collected from the health post and did not take actual adherence to preventive therapy into account. Finally, we were not able to separate the effects of the social and economic components of the intervention. To do so would have required a much larger sample size and been more expensive. In the future, larger studies could assess the differential impact of social and economic support on tuberculosis prevention and treatment and determine whether the findings are generalizable to patients with a high rate of HIV–tuberculosis coinfection or multidrug-resistant tuberculosis, patients in rural communities and those in low-income countries.

In conclusion, the socioeconomic support intervention developed in the initial phase of the CRESIPT project for application in an impoverished setting was feasible and increased: (i) the proportion of household contacts of patients being treated for tuberculosis who initiated tuberculosis preventive therapy; and (ii) the tuberculosis treatment success rate among patients. These findings highlight the need for larger-scale evaluations of the impact of socioeconomic support on tuberculosis care, prevention, control and, potentially, elimination.
